# Unsupervised underwater shipwreck detection in side-scan sonar images based on domain-adaptive techniques

**DOI:** 10.1038/s41598-024-63501-1

**Published:** 2024-06-03

**Authors:** Chengwei Wei, Yunfei Bai, Chang Liu, Yuhe Zhu, Caiju Wang, Xiaomao Li

**Affiliations:** 1 Research Institute of USV Engineering, School of Mechatronic Engineering and Automation, Shanghai University, Shanghai, 200444 China; 2 School of Computer Engineering and Science, Shanghai University, Shanghai, 200444 China

**Keywords:** Engineering, Electrical and electronic engineering

## Abstract

Underwater object detection based on side-scan sonar (SSS) suffers from a lack of finely annotated data. This study aims to avoid the laborious task of annotation by achieving unsupervised underwater object detection through domain-adaptive object detection (DAOD). In DAOD, there exists a conflict between feature transferability and discriminability, suppressing the detection performance. To address this challenge, a domain collaborative bridging detector (DCBD) including intra-domain consistency constraint (IDCC) and domain collaborative bridging (DCB), is proposed. On one hand, previous static domain labels in adversarial-based methods hinder the domain discriminator from discerning subtle intra-domain discrepancies, thus decreasing feature transferability. IDCC addresses this by introducing contrastive learning to refine intra-domain similarity. On the other hand, DAOD encourages the feature extractor to extract domain-invariant features, overlooking potential discriminative signals embedded within domain attributes. DCB addresses this by complementing domain-invariant features with domain-relevant information, thereby bolstering feature discriminability. The feasibility of DCBD is validated using unlabeled underwater shipwrecks as a case study. Experiments show that our method achieves accuracy comparable to fully supervised methods in unsupervised SSS detection (92.16% AP50 and 98.50% recall), and achieves 52.6% AP50 on the famous benchmark dataset Foggy Cityscapes, exceeding the original state-of-the-art by 4.5%.

## Introduction

Recognizing underwater objects based on side-scan sonar (SSS) suffers from few available annotated data. Constrained by the ocean’s vast expanse and the rarity of underwater objects, SSS images predominantly consist of background scenery with very few object instances. Annotating these sparse objects presents a significant challenge, being both time-consuming and labor-intensive. Moreover, diverse underwater conditions, such as turbid waters, further complicate annotating for each environment. This situation limits the volume of available data in SSS datasets.

Previous methods^1–3^ relied on style transfer to complement SSS data. However, they failed to integrate their simulation model with the detector in the training process, limiting the authenticity of simulated images. Moreover, Their detection models still relied on annotated data, restricting their applicability. In our study, domain-adaptive object detection (DAOD)^4,5^ approach is employed to overcome this problem and achieve unsupervised SSS detection. In our setting, richly annotated optical images are viewed as the source domain data, and unlabeled SSS images are viewed as the target domain data. This strategy avoids difficult SSS image annotation work through unsupervised learning.

DAOD utilizes an adversarial-based strategy to diminish the domain discrepancies between features derived from diverse domains. However, there exists a conflict between feature transferability and discriminability^6,7^: domain-invariant features are difficult to classify, which impairs final performance. In light of this, several state-of-art studies^8–10^ abandon adversarial-based training, opting instead for semi-supervised generic approaches to optimize DAOD tasks.

We identify two primary issues resulting in this conflict. First, the inability to extract entirely domain-invariant features impairs feature transferability. Previous approaches relied on fixed values to represent the domain labels, which failed to capture the intra-domain discrepancies. Adversarial training diminishes the domain discriminator’s ability to distinguish inter-domain features. The cumulative effect of these factors results in the ineffectiveness of the domain discriminator, thus impairing the domain-invariance of features. Second, prior approaches dismissed domain-relevant features which contribute to the discriminability of features. Such oversight neglects the potential discriminative signals embedded within domain attributes.

**Figure 1:**
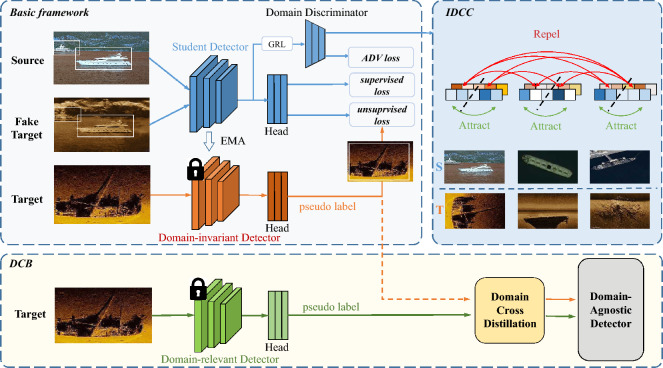
Overview of our domain collaborative bridging detector (DCBD). Training batch of the basic framework integrates labeled images from both the source and the fake-target domains, alongside unlabeled images from the target domain. The domain-invariant detector (DID), serving as the teacher, provides pseudo labels to instruct the student detector. The domain discriminator is deployed to diminish inter-domain discrepancies. Intra-domain consistency constraint (IDCC) refines intra-domain representation distribution through contrastive learning techniques. Domain collaborative bridging (DCB) leverages domain-relevant information to boost feature discriminability, yielding an optimal domain-agnostic detector.

To tackle these problems, a Domain Collaborative Bridging Detector (DCBD) is proposed to enhance the balance between transferability and discriminability. The general structure of DCBD is illustrated in Fig. 1. Specifically, to enhance feature transferability, intra-domain consistency constraint (IDCC) is proposed to rectify the vulnerabilities of static domain labels based on contrastive learning^11,12^. Through feature-level similarity comparison, IDCC encourages domain discriminators to focus on intra-domain discrepancies without supervision, thus improving the domain-invariance of features.

To improve feature discriminability, Domain Collaborative Bridging (DCB) is proposed. DCB synergizes the original adversarial-based model, referred to as the domain-invariant detector (DID), with an auxiliary domain-relevant detector (DRD) to refine features through alternating updates. DCB improves feature discriminability without introducing any additional computational burden during the inference process.

Experiments show that IDCC and DCB consistently improve the performance of DAOD, making it an effective approach to address the challenges in both DAOD and SSS image detection scenarios. Our contributions are summarized as follows:

- A novel solution: our study pioneers the realization of unsupervised SSS object detection based on DAOD. This strategy mitigates the detector’s reliance on extensive data and circumvents the laborious process of annotating SSS data. Moreover, we develop a unique DAOD dataset for the optical to SSS adaption task, comprising fully annotated optical images and unlabeled SSS images. Experiments show that the performance of DCBD closely approximates that of fully supervised training with complete annotations on SSS images.
- A novel framework: DCBD integrates the IDCC and DCB modules, adopting a ‘divide-and-conquer’ strategy to address the imbalance between feature transferability and discriminability in DAOD. Experiments show that DCBD achieves state-of-the-art performance across various DAOD tasks, effectively advancing the field of DAOD.

## Relate works

### Object recognition in SSS images

Deep learning is widely employed in the field of underwater object detection based on side-scan sonar (SSS). Early efforts^13,14^ focused on unique characteristics in SSS images, yet overlooked the scarcity of SSS data. Under the challenges posed by the limited volume of SSS data, several researchers^1–3,15^ attempted optical to SSS style-transfer techniques to enrich the dataset, thus training advanced object detectors. However, these methods still require meticulous organization and annotation of the collected data in advance. This increases the cost of SSS-based underwater object detection and restricts its applicability. Therefore, we introduce the domain-adaptive object detection (DAOD) method for SSS image detection. Our approach achieves high-precision autonomous detection of underwater objects without any pre-annotation of SSS images.

### Domain-adaptive object detection

Domain-adaptive object detection (DAOD) is designed to adapt a detector to a novel domain utilizing unlabeled data, which strives to enhance feature transferability (domain-invariance) in new domains by reducing discrepancies between feature domain distributions. However, this approach encounters a conflict between feature transferability and discriminability^6,7^: as features become more domain-invariant, their discriminability may diminish, negatively impacting the overall detection performance. Several studies seek to refine adversarial-based methods. These refinements include enhancing feature domain-invariance with additional supervision^16,17^, or bolstering feature discriminability by increasing inter-class discrepancy^18,19^.

Current industry-leading studies^5,8–10^ employed semi-supervised framework^20,21^ for further optimizing the DAOD tasks, but also encountering the imbalance between feature transferability and discriminability: in some cases, adversarial-based strategies inadvertently hinder improvements in detection performance. Consequently, some leading methods^8–10^ abandoned adversarial training, opting instead for semi-supervised generic approaches to optimize DAOD tasks. In contrast, our method refines the balance between feature transferability and discriminability at the same time, further improving the performance of DAOD based on the semi-supervised framework.

## Methods

### Basic framework

Our basic framework is based on AT^5^, it comprises a teacher model and a student model, both sharing the same structure: a feature extractor for feature extraction, a domain discriminator for distinguishing pixel-level domain labels, and a detection head for generating instance-level predictions.

The training process is divided into two phases: the burn-in phase and the teacher-student joint optimization phase. During the burn-in phase, the model is pre-trained using annotated source data and simulated target data to achieve good recognition performance. The simulated target domain image is generated by CycleGAN. During the joint optimization phase, pseudo-label techniques and consistency regularization are employed to utilize unlabeled target data, continually enhancing the detector’s performance. Meanwhile, domain discriminators collaborate with adversarial training to reduce inter-domain differences.

### Intra-domain consistency constraint

DAOD adopts static domain labels to represent distinct domain distributions, falling short in capturing the intra-domain discrepancies. Adversarial training further diminishes the domain discriminator’s ability to distinguish inter-domain information. The cumulative effect of these factors results in the domain discriminator losing sensitivity to domain information. Figure 3 illustrates this predicament, where the domain discriminator’s response to domain information becomes indistinguishable from its response to arbitrary random signals. The ineffectiveness of the domain discriminator harms feature transferability.

**Figure 2:**
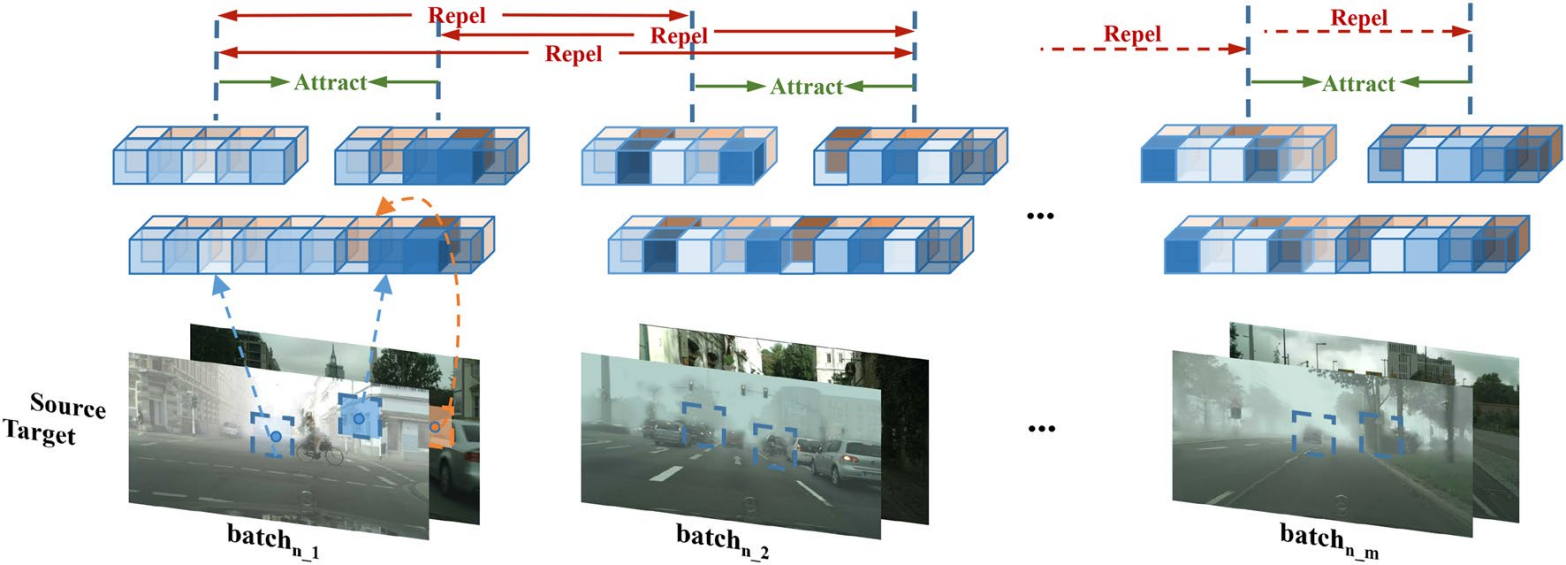
Overview of intra-domain consistency constraint (IDCC). Each image within a batch is divided into two sections, comprising different detection elements and similar domain elements. Optimization is achieved through comparative analysis of the similarity between their representations.

To tackle this, intra-domain consistency constraint (IDCC) is introduced. As precise domain labels are unachievable, contrastive learning is employed to encourage the domain discriminator to focus on intra-domain discrepancies. The logical framework of IDCC is depicted in Fig. 2.

Current state-of-the-art research, referenced as DCL^23^, introduces a decoupled contrastive learning loss that simplifies the construction of negative samples and reduces the need for large batches of samples. DCL is utilized in developing our IDCC method. In our specific task, the domain discriminator plays the role of encoder to extract domain information from each input feature. These features inherently contain a range of detection-specific information, which naturally serves as a form of data augmentation for domain information. Utilizing this, IDCC segment domain information within the same image based on spatial location to create positive pairs, and domain information from other images within the same domain is viewed as negative samples. This method sets up our contrastive learning framework.

Specifically, images within a batch of number N are processed through the feature extractor to obtain relevant features. These extracted features encompass both detection-related and domain information. Subsequently, these features are fed into the domain discriminator for decoupling and encoding, generating domain representations, which are subsequently utilized to establish positive and negative pairs for contrastive learning. Within a single image, representations are segmented into $\textrm{z}_{\textrm{i}}^{(1)}={\text {Normalize}}\left( \textrm{F}\left( \textrm{I}_{\textrm{i}}\right) ^{(1)}\right)$ and $\textrm{z}_{\textrm{i}}^{(2)}={\text {Normalize}}\left( \textrm{F}\left( \textrm{I}_{\textrm{i}}\right) ^{(2)}\right)$, creating a positive-anchor pair. correspondingly, representations from different images are segmented as $\textrm{z}_{\textrm{j}}^{(\textrm{k})}={\text {Normalize}}\left( \textrm{F}\left( \textrm{I}_{\textrm{j}}\right) ^{(\textrm{k})}\right) , \textrm{k} \in (\{1,2\})$ with k patches, and are used as negative samples. The intra-domain consistency of the domain discriminator is achieved by minimizing the contrastive loss as follows:

1 $$\begin{aligned} \textrm{L}_{\textrm{IDCC}}=-\left\langle z_i^{(1)}, z_i^{(2)}\right\rangle / \tau +\log \left( \sum _{k \in \{1,2\}, j \in [1, N], j \ne i} \exp \left( \left\langle z_i^{(1)}, z_j^{(k)}\right\rangle / \tau \right) \right) \end{aligned}$$

Consequently, the total loss for training comprises three components: detection loss $L_{\text{ det } }$, inter-domain loss $L_{\text{ adv } }$, and intra-domain loss $L_{\text{ IDCC } }$. This can be represented as follows:

2 $$\begin{aligned} L_{\text{ total } }=L_{\text{ det } }+\lambda _{\text{ adv } } L_{\text {adv}}+\beta _{\text{ IDCC } } L_{\text{ IDCC }} \end{aligned}$$

where $\lambda _{\text {adv}}$ and $\beta _{\text {IDCC}}$ are set to 0.2^5^ and 0.02 in all experiments, respectively.

**Figure 3:**
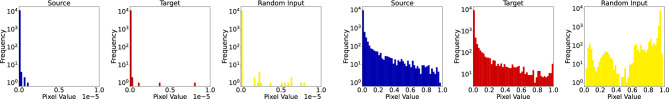
The effect of IDCC. The horizontal axis represents the output values of the discriminator. The vertical axis represents the frequency of occurrences. (**a**) Visualization of the domain discriminator’s output distribution before applying IDCC. The horizontal axis represents a value range of (0, 1e-5). (**b**) Visualization of the domain discriminator’s output distribution after applying IDCC. The horizontal axis represents a value range of (0, 1).

The visualization of the domain discriminator after applying IDCC is illustrated in Fig. 3. Owing to the additional encouragement for intra-domain discrepancies, IDCC enables the domain discriminator to maintain a positive response.

### Domain collaborative bridging

The enhancement of feature transferability tends to compromise feature discriminability. This compromise arises by overemphasizing domain-invariant features, which overlooks potential discriminative signals inherent in domain attributes.

**Figure 4:**
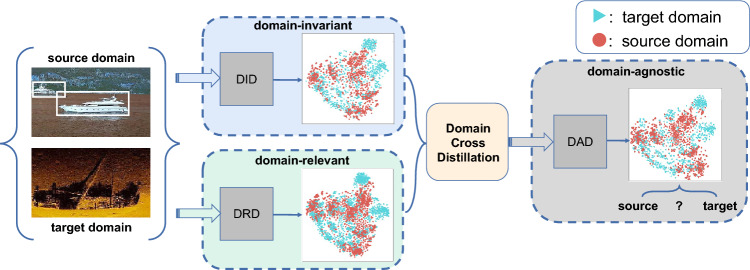
Overview of the domain collaborative bridging (DCB) approach. The associated features are dimensionally reduced and visualized using t-SNE^25^.

To address this, domain collaborative bridging (DCB) is introduced. As depicted in Fig. 4, DCB aims to complement domain-invariant features with domain-relevant information, thereby enhancing feature discriminability. This process results in a domain-agnostic detector (DAD). Firstly, we construct a domain-relevant detector (DRD) by employing domain information as additional supervision. DRD follows the same structure as the DID. For recognizing potential discriminative signals, the adversarial strategy is moved out and the feature extractor is encouraged to discern differences between domains. Let $X_s = \{x_s^i\}_{i=1}^{N_s}$ represent the source image set with $N^{s}$ samples and $X_t = \{x_t^i\}_{i=1}^{N_t}$ represent the target image set with $N^{t}$ samples, the loss of the discriminator can be represented as follows:

3 $$\begin{aligned} L_{\text {DRDdis}}=\min \left( \log D\left( E\left( x_t^i\right) \right) +\log D\left( 1-E\left( x_s^i\right) \right) \right) \end{aligned}$$

Supervision of domain information boosts the performance of DRD, making it surpasses standard semi-supervised methods. Building on this, domain cross distillation serves as a bridge between DID and DRD to harness their synergies, enabling them to teach a Domain-Agnostic Detector (DAD). DAD retains only the detection branch to maximize their complementarity. To harness insights from DRD and DID, an optimized WBF^24^ is proposed to merge their prediction based on confidence scores.

For each image, pseudo labels predicted by DRD and DID are clustered into each category based on their confidence. Bounding boxes whose intersect of union is larger than 0.6 are regarded as predicting the same object and can be fused based on their confidence. Let $\Phi ({P})$ represent a set containing N matched predictions, where ${P}^i=\left\{ {b}^i, {c}^i\right\} \in \Phi$. Here, $\mathrm {{b}}^i$ denotes the predicted bounding box, and ${\hat{c}}^i$ denotes the classification confidence. The confidence ${c}^i$ of each prediction is utilized as a weight to refine the final bounding box *b* and result in the fused pseudo label ${\hat{P}}$. This process can be formulated as follows:

4 $$\begin{aligned} {\hat{\textrm{b}}}=\frac{\sum _{i=1}^{\textrm{N}} ({\text {Max}}\left( {c}^i\right) \times {b}^i)}{\sum _{i=1}^{\textrm{N}} {\text {Max}}\left( {c}^i\right) } \end{aligned}$$

DAD is trained using annotated source data and target data with fused pseudo labels ${\hat{P}}$. Subsequently, DAD’s weights are utilized to reinitialize DRD and DID, enabling them to recover complementary capabilities without detracting from their performance. This iterative process of retraining and enhancement between DID and DRD progressively culminates in the development of a superior detector. Notably, this model introduces no additional computational burden during inference and demonstrates optimal detection performance. Supplementary Fig. S1 shows a detailed analysis of the design of the process.

## Results

### Datasets and evaluation

Following the same benchmarks as established in DAOD studies, the proposed DCBD is evaluated on the Optical Ship to Clear Water dataset. To comprehensively assess the generalizability of our algorithm, we also conduct evaluations on the following public datasets: Cityscapes^26^ to Foggy Cityscapes^27^, and PASCAL VOC^28^ to Clipart1k^29^.

**Figure 5:**
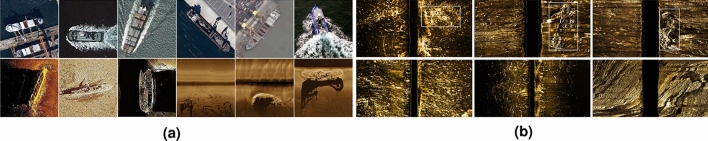
Our constructed dataset. Notably, clear water has no annotation. Turbid water is unseen in training. (**a**) Samples from the Optical Ship and Clear Water. (**b**) Samples from the Turbid Water. Instances are magnified in the white boxes for clearer visualization.

*Optical ship* comprises 856 ship images captured from optical imaging, encompassing a total of 1142 ship instances, which are predominantly characterized by a bird’s-eye view. A considerable number of open-source aerial image datasets, along with a selection of optical scene images, are utilized to construct Optical Ship. It is primarily compiled by cropping images from three aerial image datasets, namely DOTA^30^, NWPU VHR-10^31^, and NWPU RESISC45^32^, and further expanded by collecting shipwreck-like images from the internet. Image samples are presented in the first column of the Fig. 5a.

*Clear water* comprises 1635 SSS images, featuring a total of 1663 SSS images captured in clear water. Clear water is primarily compiled by open-source datasets including SeabedObjects-KLSG^1^ and SCTD^33^. Additionally, approximately 800 SSS images potentially containing shipwrecks are collected to enrich the dataset. The final dataset samples are depicted in the second column of Fig. 5a. To validate the effectiveness of our algorithm, the dataset is divided into a training set and a test set, containing 850 and 785 images, respectively. Notably, no annotations are used during the training phase.

*Turbid water* consists of 100 SSS images captured in turbid water conditions, including ten images containing shipwrecks that are difficult to distinguish. These images are collected along the Yangtze River estuary, an area where the water quality significantly impacts the visibility of submerged objects. The wooden shipwrecks in this region have undergone severe corrosion, with the majority of their structures buried in sediment, making them more challenging to identify. This dataset is exclusively utilized for qualitative experiments to ascertain the efficacy of our proposed algorithm in turbid water environments. Image samples are presented in Fig. 5b.

### Implementation details

Follows^5^, we utilize the Faster R-CNN^34^ with a ResNet-101^35^ backbone, pretrained on ImageNet-1k^36^. The weight smooth coefficient parameter of the exponential moving average (EMA) for the teacher model is set to 0.9996 and the confidence threshold for the pseudo label is 0.8. All experiments are conducted on two 3090 GPUs with a batch size 8. A fixed learning rate is maintained throughout the training stages, and the network is optimized using stochastic gradient descent (SGD). The used data augmentation methods include random horizontal flip for weak augmentation, random color jittering, gray scaling, Gaussian blurring, and cutting out patches for strong augmentations.

### Experimental settings and evaluation

We report mean average precision (IoU = 0.5: 0.95), average precision (IoU = 0.5), and recall (IoU = 0.5) in our optical to SSS experiment. Comparative analyses in other experiments are all conducted on the MS-COCO benchmark:

#### Optical to SSS

In this experiment, we evaluate DCBD on our Optical Ship to Clear Water dataset, where the object detector needs to adapt from optical imaging to underwater SSS imaging. DCBD is compared against DA-Faster RCNN^4^, as well as current state-of-the-art methods AT^5^ and PT^9^. Additionally, a comparative analysis with the fully supervised method (Oracle) is conducted in Table 1. Experiments demonstrate that DCBD stands as the most effective approach for the Optical to SSS task, achieving detection accuracy and recall rates that closely approximate those of the fully supervised method. While the DCBD produces bounding boxes of lower quality in detection tasks compared to fully supervised methods (48.64% vs 58.88% mAP), it almost achieves full recall with an impressive recall rate of 98.5% and a detection accuracy of 92.16% AP50, significantly surpassing other approaches. Experiments in Supplementary Table S2 provides a detailed ablation analysis of DCBD’s performance in the Optical to SSS task.

**Table 1:** Comparison results of the optical ship $\rightarrow$ clear water adaptation (using ResNet-101 as the backbone, with FPN) in terms of mAP, AP50, and recall.

Method	mAP	AP50	Recall
Source	9.58	21.07	54.17
CycleGAN_ICCV2017_^22^	19.45	50.37	93.64
DA-Faster_CVPR2018_^4^	10.53	26.11	56.07
PT_ICML2022_^9^	18.63	33.73	58.09
AT_CVPR2022_^5^	26.78	54.07	73.47
AT+ CycleGAN (basic)	44.92	89.20	95.52
**DCBD**	**48.64**	**92.16**	**98.50**
Oracle	**58.88**	**94.26**	97.50

Oracle represents fully supervised training.

Significant values are in bold.

#### Adverse weather

Detection across adverse weather conditions represents a classical task within the DAOD field, primarily aiming at addressing real-world application challenges. Object detectors deployed in practical scenarios frequently encounter difficulties due to varying weather conditions. Adverse weather, such as rain, snow, and fog, significantly degrades imaging quality, presenting substantial challenges to object detector performance. We evaluate DCBD on the widely-used Cityscapes to Foggy Cityscapes benchmark. The comparison results are shown in Table 2, and the detailed ablation experiments are presented in Table 3. For a fair comparison, experiments are conducted on the foggiest images (“0.02” split) within the Foggy Cityscapes dataset. The result of AT^5^ is reproduced using the released code.

Experiments indicate that DCBD achieves superior performance and even outperforms the ‘Oracle’ model. Our basic framework, incorporates CycleGAN to narrow the domain gap in the pre-training phase, yielding a 2.8% improvement in AP50. IDCC and DCB contribute an extra 1.7% increase in AP50, showing their effectiveness in adverse weather conditions. Consequently, our method not only exceeds previous state-of-the-art models by 4.5% but also the fully supervised ‘Oracle’ model by 9.4%, underscoring the substantial advancement our approach offers in domain adaptation for object detection under challenging weather conditions.

**Table 2:** Quantitative comparison results on the Cityscapes $\rightarrow$ Foggy Cityscapes and PASCAL VOC $\rightarrow$ Clipart1k benchmark.

Cityscapes $\rightarrow$ Foggy Cityscapes		PASCAL VOC $\rightarrow$ Clipart1k	
Method	mAP	Method	mAP
Source	25.6	Source	28.8
EPM_ECCV2020_^37^	40.2	SCL_2019_^38^	41.5
I-I DA_TPAMI2021_^39^	38.6	SWDA_CVPR2019_^40^	38.1
D-adapt_ICLR2022_^18^	42.2	DM_CVPR2019_^41^	41.8
D-adapt+ CycleGAN^18^	43.0	HTCN_CVPR2020_^7^	40.3
MGA_CVPR2022_^42^	43.8	UMT_CVPR2021_^8^	44.1
AT_CVPR2022_^5^	48.1	AT_CVPR2022_^5^	49.3
CMT_CVPR2023_^43^	50.3	CMT_CVPR2023_^43^	47.0
**DCBD**	**52.6(+2.3)**	**DCBD**	**51.8(+2.5)**
*Oracle*	46.9	*Oracle*	45.0

Significant values are in bold.

**Table 3:** Ablation results on the Cityscapes $\rightarrow$ Foggy Cityscapes (0.02) benchmark for each category.

Vanilla(AT)	CycleGAN	IDCC	DCB	Person	Rider	Car	Truck	Bus	Train	m-bike	Bicycle	mAP
$\checkmark$				**51.5**	49.9	61.4	36.3	57.2	49.4	37.5	45.0	48.1
$\checkmark$	$\checkmark$			47.5	48.9	65.7	42.1	61.3	57.2	39.7	44.9	50.9(+2.8)
$\checkmark$	$\checkmark$	$\checkmark$		47.3	50.5	64.9	43.9	62.4	**56.8**	40.6	**49.5**	51.6(+3.5)
$\checkmark$	$\checkmark$		$\checkmark$	47.3	**66.2**	56.9	**51.1**	61.4	42.4	**48.1**	44.6	52.2(+4.1)
$\checkmark$	$\checkmark$	$\checkmark$	$\checkmark$	48.2	51.7	**66.6**	44.4	**62.7**	**56.8**	40.6	**49.5**	**52.6(+4.5)**

Significant values are in bold.

#### Realistic to artistic

Realistic to Artistic Adaptation presents a substantial domain gap. This challenge involves pre-training models on extensive datasets to develop recognition capabilities in realistic scenes, followed by adapting these models with a much smaller dataset for artistic artwork detection tasks. We utilize Pascal VOC as the source dataset and Clipart1k as the target dataset. The results are summarized in Table 2. The foundational framework of our model contributes to a 1.2% increase in performance. Furthermore, the integration of IDCC and DCB results in an additional 2.2% enhancement in performance. These results underscore the effectiveness of our strategy in bridging the significant gap between realistic and artistic domains, showcasing the potential of our method in diverse domain adaptation scenarios.

## Discussion

We further conduct extensive ablation studies to evaluate the impact of each significant component within our model. Moreover, several studies underscore the effectiveness of our approach in addressing real-world SSS challenges and resolving the inherent conflicts in DAOD.

**Table 4:** Ablation studies on the weight of IDCC.

${\beta _{{IDCC}}}$	mAP
	Cityscapes $\rightarrow$ Foggy Cityscapes
0	50.93
0.002	50.95
0.01	51.38
**0.02**	**51.64**
0.04	51.40
0.05	51.12

Significant values are in bold.

**Table 5:** Ablation studies on the domain discriminators’ weight about DRD.

Weight of ${L_{DRDdis}}$	mAP	mAP
	PASCAL VOC $\rightarrow$ Clipart1k	Cityscapes $\rightarrow$ Foggy Cityscapes
0	47.91	50.17
0.01	49.25	50.41
0.05	50.07	50.62
0.1	50.11	50.56
**0.2**	$\mathbf {50.63}$	$\mathbf {51.06}$
0.5	48.46	50.34

Significant values are in bold.

### Ablaion study

We further investigate the effect of the designed IDCC in Table 4. An appropriate weight enhances the domain discriminator’s sensitivity to intra-domain discrepancies, thereby refining the effectiveness of adversarial training and improving the detection performance. Conversely, excessively high weight values lead to an overemphasis on intra-domain information, disrupting the balance between recognizing intra-domain discrepancies and inter-domain differences.

Table 5 shows the significance of augmented supervision on domain-relevant information for DRD. In scenarios with a substantial domain gap, such as Realistic to Artistic, additional domain supervision contributes to performance gains of 2.52%. Conversely, in a scenario with a relatively minor domain gap, such as weather adaptation, a modest improvement of 0.76% is noted. These findings suggest that domain information can facilitate the detector’s ability to recognize the potential discriminative signals, significantly influencing the performance.

**Figure 6:**
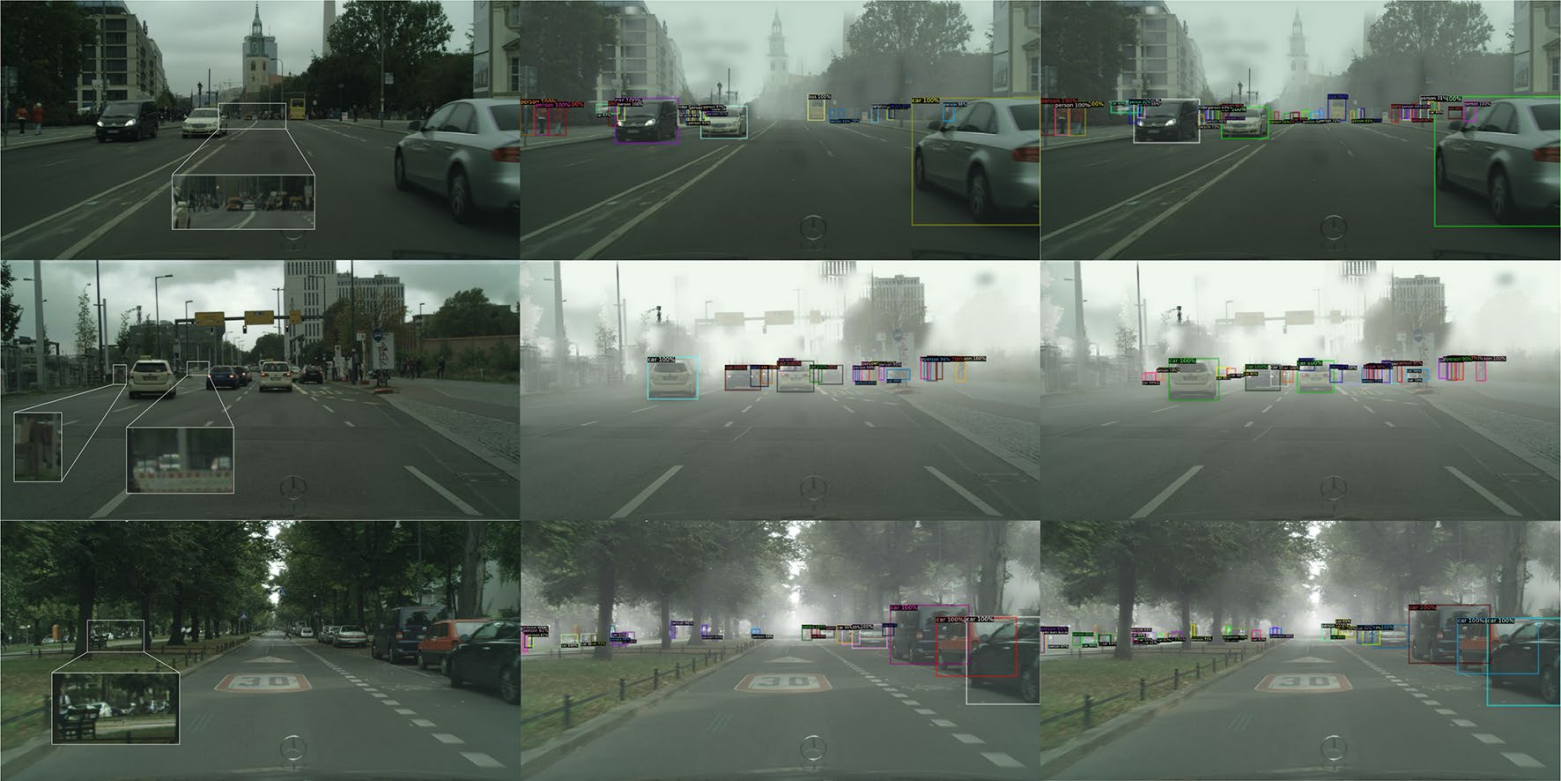
Qualitative results on Foggy Cityscapes. We compare the detection results of AT (middle) and DCBD (right) on Foggy Cityscapes. The first column displays the original images without fog. DCBD notably corrects a substantial number of false negatives (row 1, the crowd, row 2, the person and the car, row 3, the car behind the trees), effectively distinguishing instances even from distant backgrounds. Instances recalled by DCBD in the background are magnified in the white boxes in the first column for clearer visualization.

Finally, To illustrate the efficacy of our Domain Collaborative Bridging Detector (DCBD), we present some detection visualizations. As depicted in Figs. 6 and 8a, DCBD distinguishes foreground objects with greater precision and provides more accurate localization.

**Figure 7:**
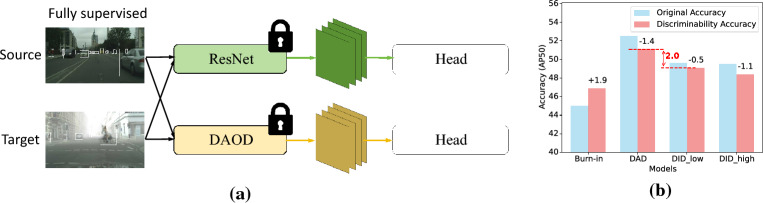
Experiments of feature transferability vs discriminability. (**a**) Feature extractor of each model is frozen and a re-initialized detection head is trained by a fully supervised method to evaluate feature discriminability. (**b**) Comparative Analysis on Foggy Cityscapes. DID_low and DID_high represent models obtained with domain discriminator weights set at 0.01 and 0.5, respectively.

### The balance between transferability and discriminability

The balance between feature transferability and discriminability is a pivotal research area in DAOD. Under consistent experimental setups, the discriminability of domain-invariant features is notably weaker compared to conventional features. Current DAOD methods based on a semi-supervised framework greatly alleviate this problem, but this inherent contradiction persists, continually impacting the performance of the detector.

Following previous research^6^, we place an investigation on feature discriminability, as presented in Fig. 7. We freeze the backbone module of the well-trained detector and employ a fully supervised approach to retrain the detection head module, aiming to assess the discriminability of the features. The results indicate that among models with considerable accuracy, models that consider domain-invariant features more demonstrate inferior discriminability (−0.6%AP50). In contrast, DCBD captures both domain-invariant and domain-relevant features, simultaneously enhancing the transferability and discriminability of these features.

### Performance on turbid water

**Figure 8:**
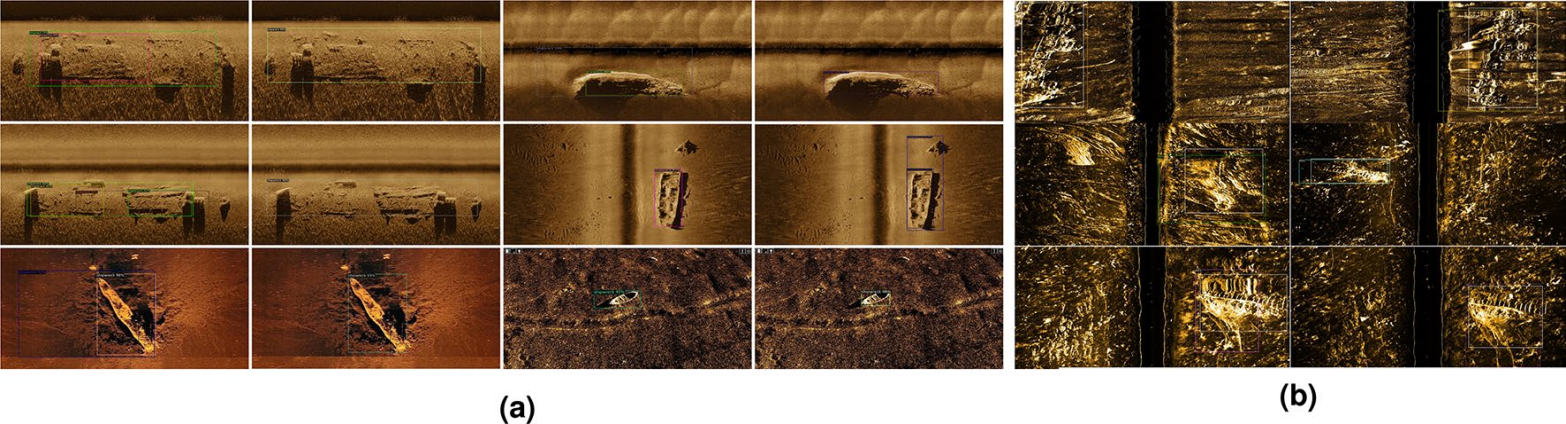
Effectiveness on unsupervised SSS detection. (**a**) Qualitative results on Clear Water. Each row displays two sets of comparisons. We compare the detection results of the basic framework (leftmost and third from left) and DCBD (rightmost and second from left). DCBD rectifies instances of false negatives, produces more precise bounding boxes (row 3, rightmost), and shows the potential to accurately recognize fragmented shipwrecks (row 2, rightmost (**b**) Visualization Results on Turbid Water. The white boxes indicate the actual locations of shipwrecks.

We evaluated the performance of DCBD on Turbid Water. Influenced by the differences in the angles of capture and the eras in which they are photographed, underwater wooden shipwrecks display varying characteristics. Visualization of the detection results is provided in Fig. 8b. The first row demonstrates the imaging features of wooden shipwrecks with minimal corrosion, partially buried in sediment yet still discernible; these are accurately detected by the algorithm. The second and third rows show shipwrecks heavily eroded and buried, where DCBD successfully distinguishes shipwreck features from the background, albeit with reduced accuracy in bounding box localization.

## Conclusion

In this study, we pioneer unsupervised side-scan sonar (SSS) object detection techniques based on Domain-Adaptive Object Detection (DAOD). This significantly enhances the applicability of object detection technology in the recognition of SSS images, as it eliminates the need for any pre-annotation of SSS images. We validated our algorithm’s effectiveness by using unlabeled underwater shipwrecks. Moreover, in the realm of DAOD, previous studies suffer from the imbalance between feature transferability and discriminability. To solve this challenge, a Domain Collaborative Bridging Detector (DCBD) is proposed, consisting of two pivotal components, Intra-Domain Consistency Constraint and Domain Collaborative Bridging. Experiments show that our method achieves an impressive 92.17% AP50 in unsupervised SSS shipwreck detection tasks, closely paralleling the precision of fully supervised methods. Furthermore, DCBD demonstrates superior performance in DAOD, achieving state-of-the-art results across multiple public datasets.

### Supplementary Information

Supplementary Information.

## Data Availability

The dataset and code of this study are available in the GitHub repository, https://github.com/firekeepers/DCBD.
