# Supplementary material for: Unsupervised underwater shipwreck detection in side-scan sonar images based on domain-adaptive techniques
Source: Sci Rep. 2024 Jun 3;14:12687. doi: 10.1038/s41598-024-63501-1 (PMC11148073; doi:10.1038/s41598-024-63501-1)
Supplement: Supplementary file 1 — Supplementary Information. [file 41598_2024_63501_MOESM1_ESM.pdf]

## Supplementary Material

### Different Strategies for Implementing Domain Collaborative Bridging

As depicted in Figure. S1, the algorithm S1a, S1b and S1c aim to utilize the complementarity between Domain-Invariant Detector (DID) and Domain-Relevant Detector (DRD) for fusion. In approach S1a, the Domain-Agnostic Detector (DAD) serves as a teacher model, guiding both DID and DRD. It updates based on the Exponential Moving Average (EMA) of the combined weight updates from DRD and DID. In approach S1b, DRD and DID function as dual teacher models. Their fused predictions guide DAD, with weights updated using EMA. Additionally, their original domain discriminator training strategies are retained to preserve their differences and complementarity. Approach S1c employs an offline update method. Initially, the network weights of both DRD and DID are frozen, and fused pseudo labels are used to guide DAD. Subsequently, the teacher models are reinitialized using DAD's weights and re-trained with their original training strategies to regain their distinctiveness and complementarity. This step can be iteratively performed to achieve optimal performance.

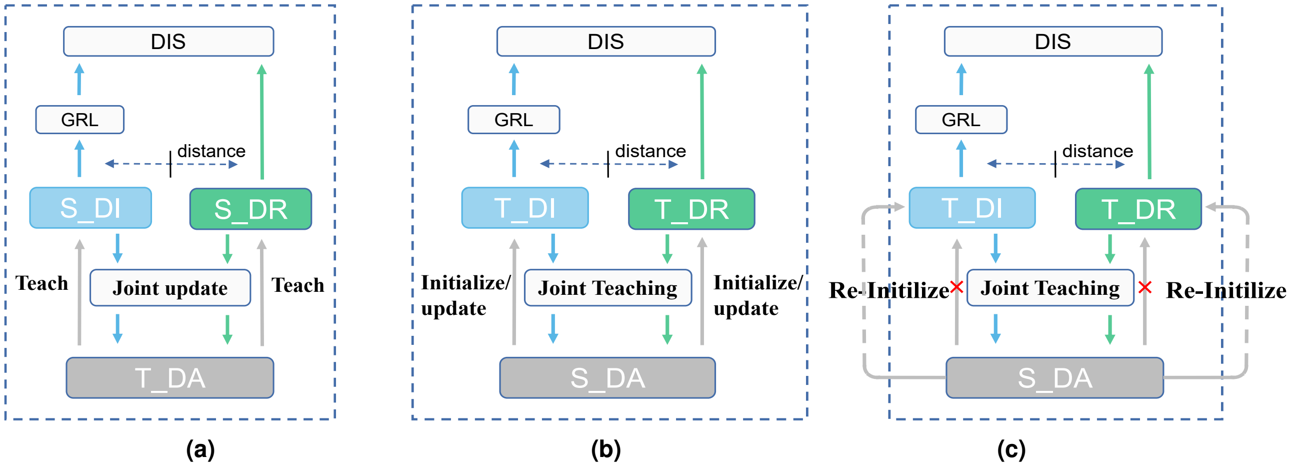


**Figure S1.** Three categories of domain collaborate algorithms. "DI", "DR", and "DA" refer to "domain-invariant", "domain-relevant", and "domain-agnostic" detectors. "T" and "S" represent the teacher and student models, respectively.

Experiments show that method S1c can be integrated into the current DAOD framework effectively to achieve superior performance. Algorithm 1 provides a detailed description of the training procedure, which is termed DCB. Additional ablation experiments are presented in Table S1.

| Method | mAP | AP50 | Recall |
|---|---|---|---|
| before | 28.0 | 50.9 | 59.3 |
| (a) | 27.4 | 50.5 | **61.7** |
| (b) | 28.1 | 51.1 | 61.6 |
| (c) | **28.6** | **52.6** | 61.6 |

**Table S1.** Comparison results of different domain collaborate algorithms on Cityscapes $\rightarrow$ Foggy Cityscapes.

**Algorithm 1:** Domain Collaborative Bridging

**Input:** training dataset: $(X_s, Y_s, X_t)$; batch dataset: $(x_s, y_s, x_t)$; teacher model: $M_{DRD}, M_{DID}$; EMA momentum: $\alpha$; alternating round: $R$; max_iterations.

**Output:** final domain-agnostic detector $M_{DAD}^{R}$.

\# alternating optimization;
**for** $r \leftarrow$ *1 to R* **do**
  EMA models initialization: $\theta_{M'^{r}_{DRD}} \leftarrow \theta^{r}_{M^{r}_{DRD}}, \theta_{M'^{r}_{DID}} \leftarrow \theta^{r}_{M^{r}_{DID}}$;
  **for** $k \leftarrow$ *1 to max_iterations* **do**
    Get source data $x_s, y_s$, target data $x_t$;
    Get fake-target images $x_{ft}, y_{ft}$;
    Optimize $M_{DRD}, M_{DID}$ ;
    Update EMA model:
    $\theta^{k+1}_{M'^{r}_{DRD}} \leftarrow \alpha \theta^{k}_{M'^{r}_{DRD}} + (1-\alpha)\theta^{k}_{M_{DRD}}$;
    $\theta^{k+1}_{M'^{r}_{DID}} \leftarrow \alpha \theta^{k}_{M'^{r}_{DID}} + (1-\alpha)\theta^{k}_{M_{DID}}$;
  **end**
  \# Domain Cross distillation;
  **for** $k \leftarrow$ *1 to max_iterations* **do**
    Get source data $x_s, y_s$, target data $x_t$;
    Get pseudo labels from $M'^{r}_{DRD}, M'^{r}_{DID}$;
    Get ensemble pseudo label $\bar{b}$;
    Optimize $M_{DAD}$;
  **end**
  **if** $r \neq R$ **then**
    Teacher models initialization for next round: $\theta^{r}_{M^{r+1}_{DRD}} \leftarrow \theta^{r}_{M^{r}_{DAD}}, \theta_{M^{r+1}_{DID}} \leftarrow \theta^{r}_{M^{r}_{DAD}}$;
  **end**
**end**

### DCBD for unsupervised SSS detection

Due to the inherent differences in imaging mechanisms and the contrasting environments of the instances captured, a substantial domain gap exists between real images and SSS images. This gap poses significant limitations on the unsupervised training of SSS images. Table. S2 details the ablation studies and intermediary processes in the training of DCBD for the optical to SSS task, emphasizing our approach's efficacy. Our experiments reveal that style-transfer and semi-supervised methods are indispensable components. By synergizing these advanced cross-domain techniques, our foundational framework pioneers in achieving unsupervised SSS image detection tasks (89.20%AP50). The integration of IDCC and DCB further refines the model, enhancing its accuracy and recall rates, and contributing to an additional performance gain of 2.96% AP50.

Moreover, we evaluate the superiority of DCBD compared to the Weighted Box Fusion (WBF)[1] method. WBF is a prevalent model prediction fusion technique that integrates outputs from multiple models during post-processing for more robust predictions. While WBF demonstrates marginally higher accuracy in the first stage than DCBD, it requires loading multiple models during inference, thereby increasing the computational burden during model deployment. In contrast, DCBD efficiently leverages the identical structures of DRD and DID along with their domain information discrepancies. Through iterative refinement, DCBD continues to enhance model accuracy in subsequent stages, achieving superior performance over WBF (3.01% mAP and 0.85% AP50).

| Components | mAP | AP50 | gain | Recall |
|---|---|---|---|---|
| Source only | 9.58 | 21.07 | – | 54.17 |
| W/o semi-supervised | 19.45 | 50.37 | – | 93.64 |
| W/o fake-target | 26.78 | 54.07 | – | 73.47 |
| W/o discriminator | 39.05 | 80.75 | – | 80.34 |
| W/o IDCC | 44.92 | 89.20 | – | 95.52 |
| Stage 1 DIDR | | | | |
| Domain-Invariant | 45.31 | 89.87 | 0.67 | 97.50 |
| Domain-Relevant | 47.78 | 90.30 | 1.10 | 98.60 |
| Weighted Boxes Fusion | 45.63 | 91.31 | 2.11 | **99.00** |
| Stage 1 DCB | | | | |
| Domain-Agnostic | 46.77 | 91.00 | 1.80 | 98.50 |
| Stage 2 DIDR | | | | |
| Domain-Invariant | 45.8 | 91.20 | 2.00 | 98.25 |
| Domain-Relevant | 46.2 | 91.61 | 2.41 | **99.00** |
| Stage 2 DCB | | | | |
| Domain-Agnostic | 46.52 | 91.86 | 2.66 | **99.00** |
| Stage 3 DIDR | | | | |
| Domain-Invariant | 46.36 | 91.50 | 2.30 | 98.50 |
| Domain-Relevant | 46.56 | 91.82 | 2.62 | 98.75 |
| Stage 3 DCB | | | | |
| Domain-Agnostic | **48.64** | **92.16** | **2.96** | 98.50 |

**Table S2.** Ablation analysis of each component and training process of DCBD in the Optical → SSS Task. 'Semi-sup' refers to the semi-supervised method. The terms 'Domain invariant' and 'Domain relevant' correspond to the 'DRD' and 'DID' models, respectively.

### Different generative methods during the Burn-in phase

Considering that using different generative methods during the burn-in phase may affect the performance of domain-adaptive object detection, we conducted an analysis of the performance based on different generative methods. We simulated underwater SSS data using CycleGAN[2], StyleGANv3[3], and Stable Diffusion Model (SDM)[4] respectively, and the results are shown in Figure. S3, S2, S4.

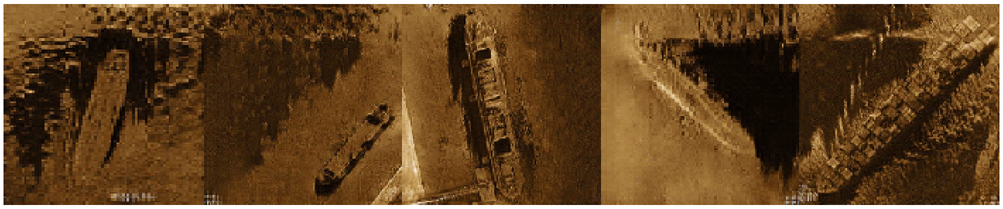

**Figure S2.** Simulated images by CycleGAN

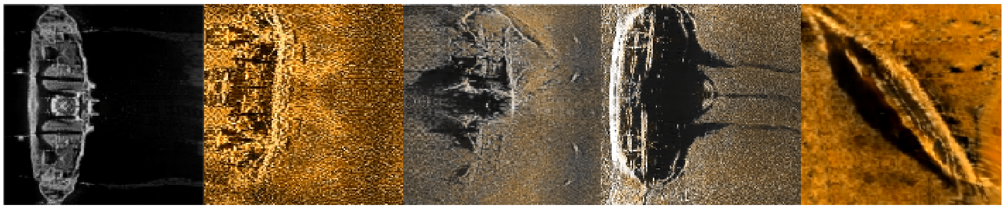

**Figure S3.** Simulated images by StyleGANv3

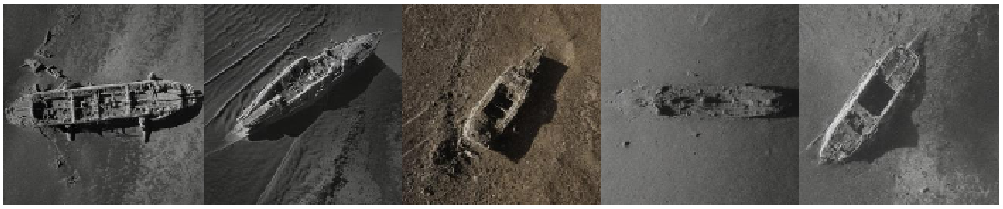

**Figure S4.** Simulated images by SDM

The simulated data generated based on SDM exhibits superior visual quality. Pre-training the model with data generated from different methods during the burn-in phase yields performance metrics as shown in Table. S3.

| Method | mAP | AP50 | Recall |
|---|---|---|---|
| Source | 9.6 | 21.1 | 54.2 |
| Source+CycleGAN | 19.5 | 50.4 | 93.6 |
| Source+StyleGANv3 | 27.7 | 57.2 | 83.6 |
| Source+SDM | 25.5 | 58.1 | 87.4 |
| Source+SDM+CycleGAN | **27.9** | **61.4** | **95.6** |
| Target | 58.9 | 94.3 | 97.5 |

**Table S3.** Comparison results of different generative algorithms during the burn-in phase.

The combined simulated data from SDM and CycleGAN yields the best results, significantly improving the model performance during the burn-in phase. Based on this data simulation strategy, the accuracy of DCBD during training is presented in Table. S4. We observed that using a better generative model can significantly improve the performance of the DRD and DID branches. However, the overall performance improvement of the DAD model was not significant, with only a 0.56% mAP and 0.01% AP50 improvement. On the one hand, this phenomenon validates the effectiveness of using SDM for shipwreck simulation, indicating that employing better data simulators can enhance the effectiveness of domain-adaptive object detectors in unsupervised underwater shipwreck detection tasks. On the other hand, the minor improvement in DAD performance indirectly reflects the superiority of our algorithm. In the absence of data simulation, the performance of domain-adaptive object detection algorithms is only influenced by three factors: the quality of optical images, the quality of sonar images, and the effectiveness of the algorithm. Assuming the use of an optimal domain-adaptive object detection algorithm, the accuracy of the detector is only affected by the quality of the images. Our proposed algorithm, which balances feature transferability and discriminability, learns domain-relevant and domain-invariant features from images captured by different branches, maximally utilizing all knowledge from optical and SSS images and achieving optimal results. Therefore, using burn-in models with higher performance only improves the stability of model training, failing to significantly enhance the model's performance further.

| Method | mAP | AP50 | Recall |
|---|---|---|---|
| DRD | 47.78 | 90.30 | **98.60** |
| DRD+SDM | **48.51** | **90.54** | 97.88 |
| DID | 45.31 | 89.87 | 97.50 |
| DID+SDM | **47.21** | **91.18** | **98.13** |
| DAD | 48.64 | 92.16 | **98.50** |
| DAD+SDM | **49.20** | **92.17** | **98.50** |
| Oracle | **58.88** | **94.26** | 97.50 |

**Table S4.** Comparison results of different generative algorithms in underwater SSS shipwreck detection task.

## Analysis of failure cases

To further analyze areas for improvement, we conducted an analysis of failure cases of DCBD on underwater shipwreck detection. Several typical failure cases are illustrated in Figure. S5: (a) Objects resembling ship masts extending beyond the boundaries of the shipwreck, lead the detector to predict a bounding box far beyond the shipwreck boundary. (b) Fragmented shipwrecks with large false shadows, lead the detector to predict multi bounding boxes. (c) False shadows resembling ship shapes, cause erroneous predictions by the detector. (d) Blank sandy landscapes.

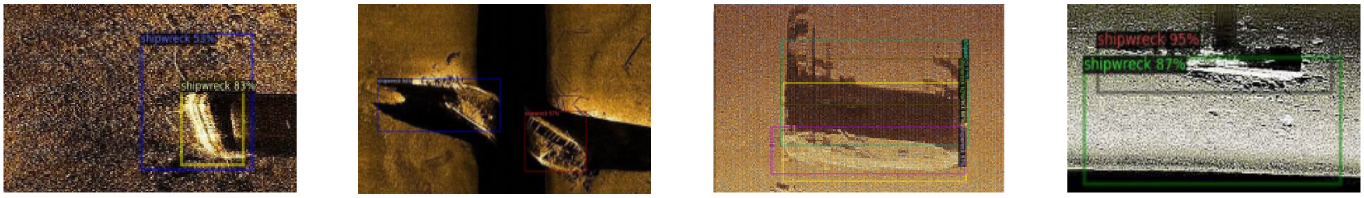


**(a)** **(b)** **(c)** **(d)**

**Figure S5.** Four categories of failure cases.

Error cases (a), (b), and (c), while lowering the evaluation accuracy of the model, don't affect the performance of the detector in practical detection tasks. Cases (a) and (b) are caused by the fragmentation of shipwrecks, making it difficult even for annotators to determine how to annotate fragmented shipwrecks accurately. For case (c), the false shadows in SSS images are an integral part of the target being detected, this failure case demonstrates the potential of Domain-adaptive object detection to bridge the domain differences between optical and sonar images while remaining unrestricted. In such cases, using accuracy to evaluate detector performance is not entirely reliable. This highlights the need for researchers to propose more effective performance evaluation methods.

Case (d) illustrates the limitations of our algorithm, where the detector misclassifies background regions as instances of shipwrecks. This deficiency may stem from two main reasons:

(1)Unsupervised training strategies: During training, the model lacks accurate SSS image labels to correct its predictions. Learning from erroneous pseudo-labels will make the detector more confident in judging incorrect predictions. This cumulative effect exacerbates detector errors.

(2)Insufficient dataset quality: The availability of bird's-eye view data in optical images is limited, which constrains both the quantity and quality of optical images. This limitation in training data directly impacts the performance ceiling of the detector.

## References

1. Solovyev, R., Wang, W. & Gabruseva, T. Weighted boxes fusion: Ensembling boxes from different object detection models. *Image Vis. Comput.* **107**, 104117 (2021).

2. Zhu, Jun-Yan & Park, Taesung& Isola, Phillip& Efros, Alexei A Unpaired image-to-image translation using cycle-consistent adversarial networks. In *Proceedings of the IEEE international conference on computer vision* 2223–2232 (2017).

3. Rombach, Robin & Blattmann, Andreas& Lorenz, Dominik& Esser, Patrick& Ommer, Björn High-resolution image synthesis with latent diffusion models. In *Proceedings of the IEEE/CVF conference on computer vision and pattern recognition* 10684–10695 (2022).

4. Radford, Alec & Kim, Jong Wook& Hallacy, Chris& Ramesh, Aditya & Goh, Gabriel& Agarwal, Sandhini& Sastry, Girish& Askell, Amanda& Mishkin, Pamela& Clark, Jack Learning transferable visual models from natural language supervision. In *International conference on machine learning* 8748–8763 (2021).
